# Diagnosis of Skin Cancer: From the Researcher Bench to the Patient’s Bedside

**DOI:** 10.3390/jcm13061523

**Published:** 2024-03-07

**Authors:** Péter Holló, Zsuzsanna Lengyel, András Bánvölgyi, Norbert Kiss

**Affiliations:** 1 Department of Dermatology, Venereology and Dermatooncology, Semmelweis University, H-1085 Budapest, Hungary; banvolgyi.andras@semmelweis.hu (A.B.); kiss.norbert@med.semmelweis-univ.hu (N.K.); 2 Department of Dermatology, Venerology and Oncodermatology, Clinical Center, Medical School, University of Pécs, H-7632 Pécs, Hungary; lengyel.zsuzsanna@pte.hu

The overall incidence and prevalence of skin cancer have shown a significant increase worldwide in the last several decades. Overall, skin cancer constitutes an important global public health issue [1]. Non-melanoma skin cancers, including squamous cell carcinoma (SCC) and basal cell carcinoma (BCC), are the most common malignancies in fair-skinned populations, leading to significant morbidity. In contrast, while its prevalence is much lower, melanoma accounts for most skin cancer-related deaths [2,3]. It was estimated that in 2020, there were a total of 325,000 new melanoma cases, and 57,000 deaths occurred due to melanoma. If these rates continue, there will be a projected rise in melanoma cases by 2040 [4]. This Special Issue, encompassing 12 publications, focuses on different aspects of the management of skin cancer, from basic science to translational and clinical studies.

Using novel biomarkers could lead to improving the diagnostics and follow-up of melanoma patients [5,6]. In the study of Várvölgyi et al., it was revealed that plasma osteopontin levels displayed a significant association with the probability of metastatic melanoma, which was similar to serum S100B levels. Moreover, this publication underlines that combining different biomarkers shows great clinical potential [7]. The role of aquaporin transmembrane proteins is intensively investigated in cancer biology [8,9]. In Camillo et al.’s study, the expression of aquaporin 1, aquaporin 8, and aquaporin 9 correlated with improved prognosis and clinical outcomes among melanoma patients [10].

Dermoscopy is one of the most important diagnostic modalities for evaluating skin cancer [11,12,13]. Sgouros et al.’s study highlights that dermoscopy has become an integral part of standard clinical practice in dermatology, based on a questionnaire completed by 366 Greek dermatologists. Their findings emphasize the need for structured training in the field of dermoscopy to promote its effective use in routine practice [14].

Innovative skin imaging techniques have also emerged in recent years, with diverse applications in skin cancer diagnostics [15,16,17,18,19]. Raman spectroscopy has a wide range of applications, such as assessing SCC, BCC, and melanoma, as Delrue et al.’s study extensively discussed. [20]. In Bozsányi et al.’s publication, a novel imaging technique, optically guided high-frequency ultrasound, has shown high sensitivity and specificity in the differentiation of high-risk BCC subtypes; thus, this technique potentially could aid clinical decision making regarding the selection of the appropriate treatment modality [21].

Rare types of non-melanoma skin cancer, such as sebaceous carcinoma, can pose a significant diagnostic challenge [22,23,24]. Cazzato et al.’s study revealed that Preferentially expressed Antigen in Melanoma (PRAME) can be useful for the subclassification of different grades of sebaceous carcinoma [25].

In addition to advancing diagnostic techniques, diverse novel treatment options have also emerged for more optimal management of skin cancer [26,27]. Ruiz-Villaverde et al. conducted a real-world analysis using the Hedgehog signaling pathway inhibitor, sonidegib, in locally advanced basal cell carcinoma. They reported significant efficacy and an acceptable safety profile [28]. Russo et al.’s publication describes a specialized algorithm for lower lip reconstruction following skin cancer excision [29]. Lázár et al.’s study emphasizes the crucial role of surgical treatment in large, locally advanced melanomas to achieve long-term disease control in addition to systemic therapy [30].

In a case report, a tattoo-associated cutaneous reaction was described in a patient receiving B-RAF and MEK inhibitor dabrafenib and trametinib therapy. This reaction could effectively be treated with topical corticosteroids, resulting in complete resolution and highlighting that suspending systemic treatment was unnecessary [31].

Special skin cancer patient populations, such as solid organ transplant recipients with cutaneous SCC, may need a more personalized approach to their management [32]. The retrospective cohort study by Salido-Vallejo et al. observed that the prognosis of cutaneous SCC is more closely related to other tumor-dependent risk factors than the immunocompromised state [33]. The retrospective analysis by Kuzmanovszki et al. revealed that elderly, polymorbid, and immunocompromised patients with cutaneous SCC can be effectively treated with anti-PD1 inhibitor cemiplimab [34].

As the Guest Editors of this Special Issue, we thank all authors for their excellent contributions. Finally, we thank the reviewers for their valuable comments and insightful feedback during the revisions.
